# Perceived Health Problems of Young Single-Person Households in Housing Poverty Living in Seoul, South Korea: A Qualitative Study

**DOI:** 10.3390/ijerph18031067

**Published:** 2021-01-26

**Authors:** Jisun Kim, Seunghyun Yoo

**Affiliations:** 1 Department of Public Health Sciences, Graduate School of Public Health, Seoul National University, 1 Gwanak-ro, Gwanak-gu, Seoul 08826, Korea; jeesun91@snu.ac.kr; 2 Institute of Health and Environment, Seoul National University, 1 Gwanak-ro, Gwanak-gu, Seoul 08826, Korea

**Keywords:** young adult health, housing poverty, single-person household, minimum housing standard, housing affordability, qualitative research

## Abstract

There is an increased prevalence of housing poverty among urban young adults; however, research on housing poverty and health is lacking. This study examined the effects of housing poverty on the health concepts of young people living alone. In-depth interviews were conducted with 20 single-person households, with individuals aged 19–39 years, living in Seoul, the demographic group with the highest housing poverty rate in South Korea. Data were analyzed using the constant comparison method. Based on the health concepts of the respondents, housing poverty negatively affected health in terms of “the occurrence and continuation of anxiety,” “the increase and continuation of lethargy,” “the difficulty in managing daily life and taking care of health,” “the lack of a dependable support person,” and “the difficulty in preventing and treating disease.” The majority of young people experienced difficulties responding to their situations, and their housing poverty was hard to overcome. This study suggests the need to expand healthy housing policies, strengthen housing safety nets, and enhance access to public resources needed for a healthy living.

## 1. Introduction

Housing is an essential component of human life and a health determinant that affects the health of individuals and society [1]. The World Health Organization (WHO) emphasizes the health effects of housing with the concept of “healthy housing”—“shelter that supports a state of complete physical, mental and social well-being” [2]. Accordingly, in 1961, the WHO Expert Committee on the Public Health Aspects of Housing presented standards for healthy housing. Since 1988, the committee has also provided guidelines promoting healthy housing policies [3]. However, a considerable number of people around the world continue to live in housing environments with inadequate water and sanitation facilities, poor ventilation, excess humidity, and a lack of air conditioning or heat [2]. For example, 9% of the world’s population lives in housing without a sanitary water supply [4]. Further, in 2016, an estimated 3.8 million people worldwide died from indoor air pollution [5].

South Korea (Korea hereinafter) achieved a 100% housing supply rate in 2001 [6], resolving the overall housing shortage. However, according to the Korean Population and Housing Census, the number of people living in inadequate housing increased rapidly from 54,000 in 2005 to 360,000 in 2015 [7]. A large proportion of this inadequate housing consists of tiny, multi-unit residences converted from non-dwelling buildings; the Korean name for these structures is “Gosiwon” (Gosiwon is the most frequent type of a formerly non-dwelling unit in Korea. Starting as temporary accommodations for students preparing for university examinations, it has become a housing alternative for single-person households. The average size of a Gosiwon is about 6.6 m^2^ (~71 sq ft), usually containing a bed and a desk; the bathroom and kitchen are shared with people living in the same building.) [8]. Housing poverty in Korea refers to a situation of living below the Minimum Housing Standard [9] or spending more than 30% of income on housing. As defined by the Korean government, the Minimum Housing Standard sets standards for room size, private kitchens and bathrooms, soundproofing, lighting, heating, and protection from noise, odor, and fire. As of 2018, 5.7% of 19.5 million households in Korea were below the Minimum Housing Standard [8]. Moreover, about 11% of all households were spending more than 30% of their income on housing [8].

According to Shaw’s comprehensive model on the relationship between health and housing, high housing costs, and poor living conditions negatively impact physical and mental health [10]. Studies based on the model have explored the relationship between housing poverty and health in Korea, finding negative impacts on health, including chronic diseases, depression, and anxiety [11,12]. Cramped living spaces are associated with increased depression, while living environments not protected from outside intrusion, noise, and objectionable factors such as bugs and odor can cause pain and anxiety. Anxiety is also associated with residential instability, and sudden rises in housing cost [10,13,14,15]. Researchers in Korea have found detrimental effects of housing poverty on residents, specifically the homeless, “Jjokbang” (Jjokbang is small room made by splitting one room into several pieces, with an average size of 3 m^2^) dwellers and the elderly living alone [16,17,18].

However, young people who move to large cities for education and employment, and who form single-person households are not considered in these discussions. Subpar housing, once used to house people moving to cities during rapid industrialization, has now become the dominant type of residence for young people coming to Seoul with low economic and social resources [19]. The 2015 Korean Population and Housing Census reported that the housing poverty rate of young households is 17.6%, compared with 11.6% among all households nationwide. The capital city of Seoul, with a large influx of young people and the highest housing costs in Korea, had a 29.6% housing poverty rate in 2015 for young people overall, while the rate for young single-person households reached 37.2%, which is more than three times higher than that of general household [7].

Research on housing and health problems of young people, especially young single-person households, has been scant internationally. However, recent studies in Korea have found that single-person households are more susceptible than multi-person households to unhealthy eating practices, mental health problems, and social health issues [20,21]. These studies, however, have rarely considered housing characteristics specific to young, single-person households.

The lack of research on the health of young people in housing poverty may be related to the traditional perspective that young people are a relatively healthy population [22]. It may also be presumed that their housing problems would be solved when they become employed [23]. However, the health of young people is deteriorating [24], and housing affordability is challenging even for the employed [23].

With this lack of research on housing poverty and health despite an increased prevalence of housing poverty among urban young adults [25], a qualitative, exploratory approach is valuable to illustrate and detail the experiences of young people in housing poverty and their health issues based on their concepts of health.

Therefore, this study aimed to describe the housing experiences of young people in housing poverty and their health perspectives, focusing on single-person households in Seoul through qualitative methods. Since the qualitative description is helpful to address the particular context that affects “facts” about the phenomena from the perceptions and inclinations of the describer [26], it was expected to contribute to achieving the specific purposes of the study, which were (1) describing the personal and social background of a sample of young people in housing poverty, (2) identifying the impact of housing poverty on their self-perceived health, and (3) exploring their strategies to lessen the health effects of housing poverty.

## 2. Materials and Methods

### 2.1. Data Collection

The main data collection method of this study was one-on-one in-depth interviewing. In-depth interviewing is suitable for deep exploration of the perceptions and experiences of research participants, and to understand their inherent meanings. It is also beneficial for understanding the backgrounds and contexts in which participants’ perceptions are formed [27].

Participants were recruited based on purposeful sampling, via an online open platform of a civic organization for the housing issues of young people in Seoul on which an explanation of the study purpose and inclusion criteria were announced. Among 52 respondents to the announcement, we recruited 20 who met the following inclusion criteria: aged 19–39 years, single household, and status of housing poverty. Housing poverty in this study was defined as living in housing that (1) does not meet the Minimum Housing Standards or (2) requires more than 30% of household income. There have been several income approach methods to measure housing cost, such as categorical, relative, ratio, and residual [28]. In this study, we chose the ratio income approach, which is used as the standard for Korean government’s housing policy, because it is clearly measured and easily understood by people [9]. Participants self-reported their housing poverty statuses.

We conducted the interviews in April and May 2020 using online video conferencing considering coronavirus disease 2019 (COVID-19) restrictions. The interviews began by introducing participants to the purpose, content, method, and process of the study, and how personal information would be protected. Informed consent for participation and recording of the interview was obtained from all responders. The participants then completed a questionnaire on demographic characteristics (gender, age, education, and occupation), health status (subjective health status, frequency of physical activity, frequency of having breakfast and of eating out, weight perception, and mental health status [including stress, social networks and satisfaction with social relationships]), and housing conditions (length of independent living, residential area, housing type and cost, internal and external environments of the home, and residential difficulties).

Initial interview questions were drafted on the basis of prior research, and an interview guide was finalized (Table 1) after conducting three pilot interviews. The semi-structured guide prompted key questions in each interview while allowing interviewers flexibility to change the order of questions or ask follow-up questions. The interviews ended by asking whether participants had additional responses or ending remarks. The average length of an interview was 73 min. The conversations were digitally recorded, and handwritten field notes were taken simultaneously. The data were transcribed verbatim within 24 h of the interview, and the field notes were incorporated into the corresponding electronic transcripts.

### 2.2. Data Analysis

We performed an inductive analysis of the interview data, where we identified interpretable units and searched for patterns and categories through iterative processes. Based on the constant comparison method [29], we read the transcripts repeatedly to perform open-coding of meaningful sentences. Once a code matrix had been created in an Excel program, duplicate codes were integrated or deleted. We next identified sub-concepts from the open-coding and created categories representing similar topics. The name of the category was made by researchers with words that encompassed the sub-concepts. Some category names were selected with in vivo coding, applying words used by the participants in the interview [29]. We then compared the categorized data with the raw materials and examined whether the organized categories adequately described the content and meaning of the data. Newly discovered data in this process were included by modifying categories, resulting in the final categories and subcategories.

To ensure the quality of the study, the preparation, data collection, and data analysis stages were based on the criteria of Strauss and Corbin [30]. To ensure consistency with existing research, we reviewed academic literature, newspaper articles, reports, presentations, and other relevant sources. For the reliability of the data, we selected participants well-suited for the purpose of the research. Researchers created a comfortable atmosphere where participants would feel safe revealing their perceptions and experiences. To achieve diversity of circumstances and universality of phenomena, varying characteristics of participants were considered in recruitment, including gender, age, occupation, and residential type and area. Furthermore, ongoing participant selection and data collection were conducted to reach a saturation point where similar statements were repeated. For validity purposes, the analysis was conducted in accordance with the steps of the constant comparison method, and the possibility of other interpretations was reviewed through iterative, repeated comparison and confirmation to minimize bias. In addition, interview results were inspected independently by two researchers and disagreements on interpretation were resolved through discussion.

To protect the anonymity of the participants, case numbers were randomly assigned during the recruitment and used for all data collection and analysis. The study was conducted with the approval of the institutional review board of Seoul National University (IRB No. 2004/003-016).

## 3. Results

The demographic characteristics of the study participants are shown in Table 2 and Table 3. Participants were evenly split between males and females, with an average age of 28.1 years. The majority (75%) was college-educated or more. Among those with a current job (*n* = 7, 35%), only two were full-time employees. Six participants (30%) were seeking work.

The participants had lived as single-person households for about 5.2 years on average, ranging from 6 months to 10 years. Studio apartments with their bathroom and kitchenette, called “one-rooms,” were the most common housing type (65%, *n* = 13). Some of the one-rooms were located in semi-basement (*n* = 2) or on rooftops (*n* = 2). The remaining participants (35%, *n* = 7) were living in varying types of collective housing where they had their room but shared bathrooms or kitchens with other residents. Monthly rental with a deposit (50%, *n* = 10) was the most common payment format, followed by a monthly rental without a deposit (20%, *n* = 4) or with a Jeonse (Jeonse is unique real estate property rights system in Korea, where it is possible to rent a house without paying a monthly rent after a substantial deposit) (20%, *n* = 4). One person was living with a short-term lease, and another person was living in a relative’s house for free. The greatest difficulties they suffered were high housing costs (40%, *n* = 8) and poor environments (40%, *n* = 8). In addition, only 20% (*n* = 4) considered themselves as healthy. Fewer than half (35%, *n* = 7) were exercising at least 10 min per day, almost half (45%, *n* = 9) did not eat breakfast, and half (*n* = 10) perceived themselves as obese. Many participants (65%, *n* = 13) felt stressed in their daily life. Regarding social networks and supports, 45% (*n* = 9) of participants did not feel that they had a social network to turn to in case of an emergency, and 30% (*n* = 6) were unsatisfied with their social relationships.

Data analysis identified 120 concepts, 47 subcategories, and 19 categories from the interviews and field notes. The categories, subcategories, and concepts were arranged and grouped according to their linkage to the research topics as well as to the interrelationships between the categories and subcategories. The resulting four topics of this study are shown in Table 4. Quotes from the participants are presented for each topic with the case IDs (Table 4) in parenthesis.

### 3.1. Personal and Social Backgrounds of the Young People in Housing Poverty

Young people entered housing poverty as a consequence of being independent without an economic foundation. For some, family conflict was a reason for their decision to move out of their family home. However, education, or employment was the main reason they left their family homes and formed single-person households (70%, *n* = 14). Seoul pulled the young respondents with better opportunities for higher education at “well-known” universities, employment, and job preparation, but adequate housing in Seoul was rarely affordable.

*“I did not have enough money to live alone independently. So I chose Gosiwon because I didn’t have to pay a deposit. I think Gosiwon is a small place hot in the summer and cold in the winter, but I would continue to live here for a while to save housing cost as much as I can. I’m working right now, with the minimum wage, I have to save as much as I can.”*

(P17)

With little budget or preparation for independent living, the young respondents had limited residential areas and housing options and ended up living in subpar housing. Almost half of the respondents (45%, *n* = 9) were spending more than 50% of their living expenses on housing, some up to 70% (Table 3), yet most were still living in residential spaces and neighborhoods they perceived as low quality and dangerous. As a result, 18 out of 20 (90%) participants were living in houses that did not meet the Minimum Housing Standard, and were “swapping money” for the proper rest, comfort, and safety they had expected “at home.”

*“I am living here because the rent and deposit are cheap... I did not know why people do not like semi-basements until I actually experienced it. It’s really moldy. The mold keeps blooming, no matter how I erase it. There are a lot of bugs as well. I live alone, but I do not feel like living alone. I have a lot of [bugs] family.”*

(P3)

*“My room is just 2pyeong (6.6 m^2^), so with a bed, desk, refrigerator, and book shelf, there is very small space left. Therefore, I cannot invite anyone in my room. I spend almost all time on the bed when I am at home because there is no space to move.”*

(P17)

The respondents desired to move out of their current residences and transition to Jeonse-type housing with better quality and no monthly rent burden. Jeonses, however, require a large, one-time payment upfront, so affordability is even more challenging. Persistent unemployment worsens the situation where young people have difficulties in finding jobs, even for part-time. The majority of respondents believed that even with employment, it would be difficult to save enough money to move to a Jeonse while paying a monthly rent with low wages. They also mentioned that getting loans might not be feasible since many jobs were currently insecure. Moreover, the COVID-19 pandemic had canceled many jobs and reduced work opportunities for young people.

*“It was really difficult to get even a part-time job. I was struggling with the employment shortage and barely managed to find a part-time daily wage job. It wasn’t enough to pay the rent. So I use emergency loans to pay the rent almost every year.”*

(P15)

*“I have to get a job again, but it is not easy because of COVID-19. There aren’t many job openings. But I really need to find a job soon because now I’m living on savings. I have almost run out of money now.”*

(P4)

When both students and the employed experience unaffordability of adequate housing, families have been regarded as the main source of support. However, 13 of the 20 participants (65%) could not receive support from their families who were also financially challenged. These young people sought support from friends and others in their personal network. However, some with limited networks did not have someone to turn to, and some were already in debt to their friends.

After family support for housing assistance, the next most common choice was the public sector housing support program. However, only 5 out of 20 (25%) participants had utilized public support for housing. More participants had approached the public support program but found that the eligibility was specific to a certain group of young people. Some of those accepted to public housing program were forced to give up the opportunity because they could not pay the required minimum deposit. Others who were fully occupied with making a living did not have enough spare time to learn about the programs and prepare the necessary applications.

*“For the government-funded housing programs, I was not a university student, not an office worker, I did not belong anywhere in that category of the eligible. And, even if I get accepted to a public rental house, I need to pay deposit, but I do not have any money. So even if I am accepted, I cannot get in.”*

(P11)

### 3.2. Health Concepts of the Young People in Housing Poverty

The most salient aspect of the perceived concept of health among the study participants was “being mentally stable.” In describing their health concept, most explained mental stability as reducing negative issues, such as “less anxiety and stress.” A smaller number of respondents linked mental stability with positive images such as affirmation of one’s self. Their anxiety was associated with the idea that they will not meet their social expectations, such as having a job with a certain guaranteed level of income and employment security, owning a house, and eventually having a family.

*“I think I will be healthier in my 30s. I am preparing for an exam and am anxious about many things. If I pass the exam and get a job, the anxiety will be gone and I will be much better than I am now. Relationships with people will get much better then.”*

(P14)

To the participants, health was necessary for what they have to do and what they want to do well. Studying and preparing for employment, in particular, were considered as main tasks to accomplish at their age, and health was recognized as an essential factor for succeeding in those tasks.

*“Young adulthood is a period when I am building the foundation for my life. If I am not healthy, it would be so difficult to accomplish what I’m supposed to accomplish while I’m young. … I think we lose time because we are unhealthy so much. I think time is the only strength we have, so being healthy means we have a weapon to fight with.”*

(P5)

Health was also important for the participants “to make a good daily life.” They perceived that an enjoyable daily life involves getting enough rest and adequate sleep, eating healthy food regularly, waking up well in the morning, and being comfortable with the people they meet. That perception came from their experiences of feeling unhealthy when they could not manage daily life well.

*“Working and resting enough after work, taking a walk, having some snacks with people I like... If I can maintain those activities in my daily life, I think I am healthy and live well. Of course, that is not easy.”*

(P16)

The existence of a dependable support person” was another component of perceived health. The respondents valued having someone to rely on, share their daily life and innermost thoughts, and provide help when needed as part of their health. Without a supportive person, they felt that they would be isolated and anxious.

*“It is very important to me to have supportive relationships with people around me. Even when I was sick or tired, I experienced the situation differently depending on who was around me and whether the person was safe to share my feelings with. If there is a person around me I can rely on, I think I am healthy.”*

(P4)

Some of the respondents viewed “the absence of disease” as health. Most were currently seeing physicians or taking medications for health conditions, hoping for recovery through treatment.

*“Health... is not getting sick anywhere from fingertips to toes when I sit still. Because my back hurts, I have been to the hospital a lot, and I am still going...”*

(P7)

### 3.3. Health Effects of Housing Poverty

The participants identified and described the health effects of housing poverty on the basis of their perceived health concepts. Given that mental stability was perceived as health, anxiety caused by housing poverty had negative health effects. Young people in poor housing conditions were always exposed to security issues, including crime and fire risks. The anxiety over housing costs, in particular, emerged in three aspects: not being able to pay the monthly rent, facing a sudden increase in housing cost, and failing to get their deposit refunded. The anxiety about housing costs and financial instability led to anxiety about resolving their housing poverty. One young man expressed such anxiety with a remark, “I should not be homeless.”

*“I am living in a semi-basement. When I look out the window, especially the kitchen window, I directly see the sidewalk where people pass by. If someone drops something and ducks to pick it up while I’m washing the dishes, I make eye contact with him. I cannot open the window because I feel so insecure.”*

(P3)

*“It may sound negative, but all I want is not to be homeless. I do not want to be in a more difficult situation than now. In fact, I was on the verge of becoming homeless. I did not have any money in my bank account.”*

(P7)

As for health, including having enough physical strength to do what one needs and wants to do, many participants evaluated their physical strength as poor. They felt lethargic due to poor housing conditions—confined rooms, limited living space, and poor ventilation—and they were financially restricted from finding something to overcome the lethargy.

*“I am living in a rooftop one-room [studio] where the kitchen and living room are not separated [from the bedroom]. It is just a space with four walls, each of which has a door—to bathroom, front door, and windows. It feels like I’m always floating trapped in this confined space, and I have no energy to do anything in the room.”*

(P10)

The low-quality housing where the participants resided was largely in disadvantaged neighborhoods, thus they were restricted in resources and opportunities to be healthy. Having a healthy diet was difficult without a kitchen or in neighborhoods without access to fresh foods. Small and confined rooms were often too small to do stretching exercises, and neighborhoods that lacked green space or parks also limited active living. Living in a room with insufficient soundproofing exposed the residents to constant noise.

*“Because of the noise from the bar on the first floor of the building where I am living, I suffered a lot last year from nightmares. Also, I woke up a lot in the middle of night. I could not fall asleep until 5 o’clock in the morning, when the bar closed.”*

(P19)

Regarding the concept of health, including being in dependable relationships with regular contacts or nearby support sources, participants experienced a sense of disconnection and isolation. The potential lack of support in an emergency contributed to elevated anxiety. The prolonged COVID-19 situation further reduced the frequency of meeting friends and made participants feel even more lonely and isolated.

*“What if I die home alone? I get anxious that no one would find me. Water leaks in my house when it rains, especially near the fuse box. One time I got shocked by electricity, then I thought, if I die like this, I would only be found when the rent is overdue.”*

(P2)

Unsanitary living conditions caused by housing poverty posed a threat of disease to young people. Forty percent of the participants experienced health problems caused by mold in humid and difficult-to-ventilate environments. They reported negative respiratory effects such as sore throats and stuffy noses. Some suffered from frequent infestation of pests such as cockroaches in dark and humid rooms or from neglected garbage outside of the building.

*“I have a sore throat because of the mold. There were times, not along ago, when I could not even speak.”*

(P13)

*“In the one-room I live in, the oil particles seem to float in the air after cooking. It is like I inhale the oil because the ventilation in the room is poor. It feels like my health is going bad.”*

(P20)

Although some participants suffered from diseases that require regular treatment, due to the burden of healthcare expenses, they sought care only when they had “deadly” pains. Healthcare was on the back burner for young people at the verge of job loss and wage cuts, and for whom “fixed but essential-to-life” housing costs were burdensome.

*“I did not have any money to go to the hospital because paying monthly rent is hard enough for me. So I did not go despite being in pain. In fact, I hurt my wrist. The doctor said it would be okay in two weeks. But I could not continue the treatment and now it has become chronic.”*

(P15)

### 3.4. Response Strategies to the Health Effects of Housing Poverty

The participants were trying to protect their health by making the best of their current living environment as much as possible. Those with a small kitchen, for example, tried to eat regularly by “cooking at least one meal a day for themselves.” Even in cramped rooms, some practiced home-based training facilitated by online videos.

*“I did not like to exercise, but nowadays, I think I really need to exercise because I do not feel good. So recently, I have been doing a little bit of home-based exercise with online video instructions. It is not easy though because my place is so small...”*

(P8)

Some worked to relieve loneliness and anxiety by sparing time for hobbies such as writing, drawing, reading, watching videos, and baking. Others turned to “escape sleep.”

*“When I feel really lonely, I try to fall asleep. In sleep, I meet people. I can go anywhere in dreams. It is an escape dream. It is an escape sleep. Sometimes, I dream in my dream. It is like I’m escaping from a tough situation through dreams.”*

(P7)

Improving the current living space was another strategy for having a good daily life. Cleaning with mold remover, dusting, and frequent ventilation were examples of efforts to make their homes pleasant and prevent health problems. However, some participants eventually gave up trying to eliminate mold and pests because they could not afford new wallpaper or professional pest control services. An external response strategy was to utilize local public infrastructure as well as personal resources and networks. Local libraries were pleasant and resourceful places to be instead of their confined rooms. Playgrounds and walking paths provide space for regular exercise. Those experiencing difficulties due to severe anxiety and stress utilized public counseling services designed for young citizens.

*“I am so nervous because I am without a job now. While I was receiving getting unemployment benefits, I had counseling from [the public service] for free. Since the free services ended, I looked for opportunities for free counseling.”*

(P12)

Some participants would pay out of their pockets to utilize non-public resources to cope with challenges or be in a different environment other than their homes. Local cafés were common choices for escape, while joining a gym was an example of spending scarce personal resources to stay healthy. Those for whom public counseling programs were insufficient visited private counseling services. However, going to cafés and gyms was unaffordable for many participants. Psychological counseling was even more expensive; thus, many participants reduced the frequency of their visits or eventually stopped.

*“When I am depressed, being in this place feels like I’m dazed in outer space. In a bigger place you can refresh yourself just by going from room to room, you know. But it is difficult to get out of a depressing mood in my small one-room. That is why I go to cafés in my neighborhood. But the problem is, I cannot go there often because of the cost.”*

(P2)

Another coping strategy was to be part of human networks. They joined single-household communities or hobby groups, such as book clubs, to relieve their loneliness and anxieties. Some were able to talk to close friends to shake off gloomy feelings. However, there were some young people who did not have friends to contact easily.

*“I do not have many opportunities to make new friends. In fact, I do not have any friends. I became sensitive after having tough times for several years, and it is difficult for me to get along well with others. Now I do not have the time and money to make new friends, and I am not sure who I would call if something happens to me.”*

(P18)

Among all things, the young people regarded moving to a better place as the fundamental solution for their health problems. However, most of the participants, including those employed with income, found it difficult to save enough money to move to a better place. They thought it was important to make good use of public housing opportunities; however, many were not eligible because the policy was tailored to married and child-bearing households.

*“The reality of my generation is different from that of the previous generation. The proportion of single-person households is increasing, and co-residence with people who are not their family is common now. However, the housing policy is still designed for four-member families, so there is a great gap between reality and policy.”*

(P12)

With the fundamental solutions currently unfeasible, participants had virtually no expectations of escaping from the current housing situations. They strongly believed that their low salaries, unstable employment, and poor living conditions would not change in the near future. Accordingly, they also negatively predicted their future health. Many believed that they would continue to live as single-person households. Particularly frustrated were those pessimistic about getting married. They felt they would not be able to break away from the single-person household because they do not have the financial assets to “buy a house.”

*“I will be at the same economic level and my wage will be the same in years to come. It will not change dramatically. So I do not think my health is going to change that much from now.”*

(P9)

*“People like me cannot get married because literally I cannot afford a family home. I do not want to live alone for the rest of my life, but I cannot get married…”*

(P13)

## 4. Discussion

This study explored the personal and social backgrounds of young single-households in housing poverty living in Seoul and their strategic response to perceived health problems associated with housing poverty. Young adulthood is considered a transition period from childhood to adulthood through achievements, such as graduation, employment and, marriage. However, in recent times, accomplishing these achievements has become more difficult and takes more time than earlier and is even likely to fail [31]. In this “frozen transition” [32], young adults who rely solely on their own resources increasingly have negative life and health trajectories. Therefore, the need of social support for their health is of increasing concern [33].

Young Koreans are no exception to this global trend, and most of our respondents linked poor health to negative experiences. In particular, more than half of the participants perceived health as “being mentally stable,” meaning that they felt mentally unstable from anxiety and stress. In addition, they thought that health was not something that should take time to pursue, but should be the result of the way they “have a good daily life” with tasty and nourishing food, enough rest, and a dependable support system. Therefore, focusing on their daily activities and providing support to young people at risk of activity disruptions would help them live healthy lives [34].

Meanwhile, for young people expected to carry out life tasks, moving to big cities is not an option but an essential process, as cities have more opportunities for high-quality education and employment [35]. However, those who move immediately face a housing problem involving high costs [36]. The options for living space for people with few or no assets were relatively inexpensive but of poor quality [19].

Our results showed that poor housing conditions and the burden of housing costs had negative impacts on our young respondents’ health. They suffered from unsanitary living spaces and a lack of community resources, with both direct and indirect negative effects on their physical and mental health, as reported in prior studies [37,38,39]. Moreover, the burdensome housing costs made them anxious and stressed, foregoing things necessary for their health like medical care and healthy food [40]. Some were socially isolated as single-person households [41,42], with no one to ask for help in an emergency.

Especially, housing poverty negatively impacts the mental stability of young people. These findings were consistent with prior research that states narrow residential spaces with no minimum area guaranteed would increase depression [43], an unprotected residential environment, such as intrusion from the outside and noise, would cause pain and anxiety [14], and that the instability of settlements experienced as tenants and sudden increase in housing costs would increase anxiety [13,23].

Several participants of this study had experienced housing poverty as a single-household for many years and felt trapped in their current status. This means that the housing poverty for young people should no longer be treated as a temporary condition, as represented by the word “Generation Rent,” limited to young adults (18–40 years old) unable to buy a house or pay high housing costs [44]. We found that young people were not as healthy as expected owing to conditions associated with housing poverty. Some of them were at risk of health deterioration in the long-term without any alternatives to improve their housing conditions. Furthermore, housing problems affect not only health but various areas of life such as study, employment, marriage and family planning, and interpersonal relations [45]. With access to quality, affordable housing becoming extremely difficult in cities [46]. We found that though cities can provide opportunities for young people, they, at the same time, can be unhealthy places to live.

To make cities healthy for young people, providing affordable and decent housing is essential. For example, the National League of Cities, representing 19,495 cities in the United States, suggests “healthy affordable housing policies” with specific action plans to expand affordable housing stock and improve housing quality [47]. This demonstrates that the public health perspective could be added to housing policies, as well as the further need to collaborate with other disciplines such as social welfare, sociology, geography, architecture, and urban planning [48].

In addition, we found that the built and natural environments and public services are also important for the health of young people in cities. Our participants looked for ways to overcome their housing limitations through community-based options to improve their health. However, the quantity, quality, and accessibility of public resources such as public services, green areas, and parks were scarce in the regions where many of them lived. Public resources differ across regions, and communities with high-quality housing tend to have more resources [10,49]. Thus, public planners must check the quantity, usage, and quality of public resources in areas where many young people live when developing and implementing improvements.

Furthermore, city-dwelling people perceived themselves as being lonely and anxious with, in many cases, family and established friends living far away. In fact, people in cities spend more time alone [50]. Not everyone suffers from loneliness, but when loneliness does become severe, mental health problems or even mental illness can develop [51]. This study found that young people on the “long and twisting path to adulthood” [52] felt isolated as they were not following the life path expected by society. Therefore, addressing problems associated with urban isolation should be a public health priority, with policy interventions at the individual, community, and societal levels [51].

Although this study contributes to understanding the health of young people suffering from single-person household housing poverty, the focus of the discussion is on the big picture and does not consider differences such as gender and age group in detail. Further research is needed to consider those differences. This study also used ratio-income approach to define housing poverty; however, future studies need to use more than one approach to identify various causes of poverty. Despite this limitation, this study provides basic data to set course of public health policy needed for young, single-person households facing housing poverty.

## 5. Conclusions

This study aimed to address housing as a determinant of young people’s health, while drawing implications for establishing policies to promote the health of those affected by housing poverty. Using qualitative methodology, efforts were made to capture the perception and experience of health problems related to housing poverty. Most young people who participated in the study linked health with a stable mentality, but their difficulties in everyday life due to housing poverty made them unstable.

Through this study, we found that health problems of young single-households in housing poverty living in metropolitan areas are at the intersection of the problems of the young generation—trying to carry out life tasks while living in expensive but poor quality housing. With the emergence of “Generation Rent,” it has become a complicated problem of cities worldwide, relating not only to the health of young people but also to other areas such as housing policy, social welfare, and urban planning. Therefore, from the public health perspective, collaborating with other disciplines to employ public resources and policy interventions to ensure healthy and affordable housing is critical to promoting young urban dwellers’ health.

## Figures and Tables

**Table 1:** Interview Questions.

Topic	Questions
(1) Background of current housing situation	How did you come to live in your current house/room? Please describe the housing you have previously lived in as a single person, and the place you currently live in. Please explain any difficulties you have with your current residence.
(2) Concept of health and the health effect of housing poverty	Please describe what health means to you. Please explain what is important for you to live a healthy life. How does your current residential environment differ from the previous ones? How does your current residential environment affect your health?
(3) Response to health effects of housing poverty	How are you dealing with the issues in your housing and living environment while trying to lead a healthy life? What do you anticipate your living and health situations will be like when you transition from your 20s to your 30s (or 30s to 40s), and why?

**Table 2:** Results of Questionnaire.

Variables		Count	%
Legal Sex	Male	10	50
	Female	10	50
Age (yrs.) Average: 28.1 Range: 19–37	19–29	12	60
	30–39	8	40
Education	High school and below	5	25
	In college	5	25
	College degree	6	30
	In graduate school	4	20
Occupation	Freelancer	2	10
	Part-timer	3	15
	Full-timer (office worker)	2	10
	Jobseeker	6	40
	University or graduate students	7	25
Length of independent living as a single-person household (yrs.) Average: 5.2 Range: 1–10	<3 years	6	30
	≥3 years, <6 years	5	25
	≥6 years	9	45
Residential Type	Studio Apartment (with own bathroom and kitchenette, called ‘one-room’)	13	65
	Collective housing (own bedroom with shared bathroom or kitchen)	7	35
Rental Type	Monthly rent with a deposit	10	50
	Monthly rent without a deposit	4	20
	Jeonse	4	20
	Short-term lease	1	5
	Free of charge	1	5
Rent to Income Ratio (%) Average: 36.2 Range: 0–70	<30%	7	35
	≥30%, <50%	4	20
	≥50%	9	45
Major Residential Difficulties	Over-burdened housing cost	8	40
	Poor environment (small space, poor soundproofing, unsafe security system, etc.)	8	40
	Sense of isolation	3	15
	Anxiety about housing contract	1	5
Subjective Health Status	Very Good	1	5
	Good	3	15
	Moderate	9	45
	Bad	5	25
	Very Bad	2	10
Physical Activity (exercise at least 10 min/d)	Yes	7	35
	No	13	65
Frequency of Breakfast (per week)	0	9	45
	>0, ≤3 times	5	25
	>4 times	6	30
Weight Perception	Perceived as obese	10	50
	Perceived as moderate	9	45
	Perceived as thin	1	5
Stress in daily life	Very high	5	25
	High	8	40
	Moderate	5	25
	Low	2	10
Existence of social network needed in emergency	Yes	11	55
	No	9	45
Satisfaction with social relationships	Satisfied	10	50
	Moderate	4	20
	Unsatisfied	6	30
Total		20	100

**Table 3:** Participant Characteristics.

Case ID	Legal Sex	Age	Occupation	Education	Length of Independence (yrs.)	Residential Type	Rental Type	Rent to Income Ratio
P1	M	31	Student	Graduate Student	5	Studio Apt.	Monthly rent	37
P2	F	28	Job Seeker	University Graduate	10	Studio Apt.	Monthly rent	50
P3	F	32	Freelancer	Graduate Student	1.3	Studio Apt.	Jeonse	20
P4	F	31	Job Seeker	University Graduate	6	Studio Apt.	Jeonse	30
P5	F	22	Student	University Student	1.2	Lodging	Monthly rent	50
P6	M	25	Office Worker	High school Graduate	5	Studio Apt.	Monthly rent	70
P7	M	34	Student	Graduate Student	10	Studio Apt.	Monthly rent	50
P8	F	25	Office Worker	University Graduate	9.5	Studio Apt.	Monthly rent	25
P9	M	26	Freelancer	University Graduate	6.1	Share House	Monthly rent	30
P10	F	25	Job Seeker	University Graduate	8	Studio Apt.	Jeonse	10
P11	F	23	Job Seeker	High school Graduate	3.8	Studio Apt	Monthly rent	50
P12	F	34	Job Seeker	High school Graduate	7.1	Gosiwon	Jeonse	20
P13	M	36	Part-timer	High school Graduate	8	Studio Apt.	Monthly rent	50
P14	M	23	Student	University Student	1	Dormitory	Monthly rent	10
P15	F	28	Part-timer	High school Graduate	5.5	Guest House	Short-term lease	70
P16	M	19	Student	University Student	1	Relative’s House	Free of charge	0
P17	M	32	Part-timer	Graduate Student	9.3	Gosiwon	Monthly rent	12
P18	F	37	Job Seeker	University Graduate	5.4	Studio Apt.	Monthly rent	60
P19	M	26	Student	University Student	8	Studio Apt.	Monthly rent	30

**Table 4:** Results by category and subcategory.

Topic	Category	Subcategory
Personal and social contexts of living in Seoul	Being independent without an economic base	Coming to Seoul for study and employment
		Sudden independence due to family problems
	High housing costs and poor housing conditions in Seoul	Expensive housing costs
		Poor living environments and conditions
	Wages not equal to housing costs	Shortage of jobs
		Low-paying jobs
	Lack of private and public resources	No financial support from the family
		Difficulty asking for help from friends or acquaintances
		Difficulty in utilizing public housing policies
The health concept of the young people in housing poverty	Being mentally stable	Less anxiety and stress
		A positive mind
	Sufficient physical strength	Physical strength to do what needs to be done
		Physical strength to do what one wants to do
	“To make a good daily life”	Sufficient sleep and rest
		A regular and balanced diet
	Existence of dependable support person	Someone who can share my values and interests
		A person that can help me in a crisis
	Absence of disease	Not being sick at this time
		Not being sick in the future
The effect of housing poverty on health	Occurrence and continuation of anxiety	Anxiety about public security
		Anxiety about fire
		Anxiety about housing costs
		Anxiety about the permanence of housing poverty
	Increase and continuation of lethargy	Growing lethargic in a confined room
		Constraints of activities that could help overcome lethargy
	Being tough in leading a daily life and taking care of one’s health	Problem in sleep and rest
		Difficulties in exercising regularly
		Less opportunity for healthy eating
	Lack of dependable support person as single-household	Absence of daily social contacts
		No one to seek help from in an emergency
	Difficulties in preventing and treating diseases	Unsanitary environment that can cause diseases
		Going without medical treatment due to housing costs
The response strategy to the health effects of housing poverty	Try to “have a good day” alone in the house	Regular meals and exercise
		Activities to relieve loneliness and depression
		Making living space comfortable
	Looking for alternatives outside of the house	Utilization of public resources
		Utilization of private resources
		Utilization of social networks
	Planning and expecting changes in the current housing situation	Change of housing conditions by moving
		Formation of a multi-person household
	No expectations and alternatives for changing the situation	Continuation of housing insecurity
		Remaining a single-person household

## Data Availability

The data presented in this study are available on request from the corresponding author. The data are not publicly available to protect confidentiality of the research participants.
